# Sulfur dioxide emissions curbing effects and influencing mechanisms of China’s emission trading system

**DOI:** 10.1371/journal.pone.0276601

**Published:** 2022-11-09

**Authors:** Fengge Yao, Lin Li, Shen Zhong

**Affiliations:** School of Finance, Harbin University of Commerce, Harbin, China; Yunnan Technology and Business University, CHINA

## Abstract

The emissions trading system, a crucial and fundamental system reform in the environmental resources field of China, was established to promote the continuous and effective reduction of total emissions of major pollutants. In this context, based on the panel data of 285 Chinese cities (except Tibet) from 2004 to 2018, this paper uses the quasi-experimental method of Difference in Difference to assess the effect of the emissions trading system introduced on sulfur dioxide emissions of China and the transmission mechanism. The article generates several intriguing findings. (1) The emissions trading system has a significant suppressive effect on sulfur dioxide emissions. (2) Mechanistic tests show that the emissions trading system can effectively suppress sulfur dioxide emissions by reducing government intervention, stimulating green patent innovation, and improving resource use efficiency, in which green utility patents have a masking effect. (3) From the east, central and west divisions, the emissions trading system has a significant suppression effect on sulfur dioxide emission in the eastern and central regions, and the eastern region is better than the central region. (4) In terms of factor endowment, the emissions trading system has a significant suppression effect on sulfur dioxide emissions in both resource-based and non-resource-based cities, with non-resource-based cities outperforming resource-based cities; while within resource-based effect exists only in regenerative cities. (5) The emissions trading system has a significant suppression effect on sulfur dioxide emissions in old and non-old industrial base cities in industrial base zoning. The suppression effect in non-old industrial base cities is better than that in old industrial base cities. This paper provides empirical evidence for evaluating the emissions trading system at the provincial level in China and suggests policy recommendations for selecting government tools to effectively curb sulfur dioxide emissions. Although the emissions trading system has made an outstanding contribution to sulfur dioxide emissions reduction, there is still much space for further development of potential emission reductions.

## 1 Introduction

As one of the major air pollutants [1], sulfur dioxide (SO_2_) emissions are the main cause of acid rain, fog, and haze [2], which pose a great threat to economic development, human health and ecosystem security [3]. In the long run, the deterioration of economic performance due to the growth of SO_2_ emissions will overshadow the enhancement of economic performance under this status quo [4]. The phenomenon of environmental pollution caused by excessive emissions of SO_2_ is regarded as a phenomenon with negative externality characteristics, and in the face of such negative externality problems, it is particularly important to take control measures on the emitters [5], especially the use of climate policy tools [6]. In addition, Coase’s theorem is a theory used to analyze transaction costs and their relationship with property rights arrangements. The idea points out that due to transaction costs, different definitions and allocations of rights lead to other efficient resource allocations. Dales J. H. (1986) applied Coase’s theorem to point out that pollution is a property right given by the government to the emitting firm, and this discharge right can be transferred by market means so that market instruments can be used to improve the efficiency of environmental pollution control [7]. However, in the comparison of the strength of the role of public awareness and government awareness, the government has more influence than the public and industrial enterprises, so the government should intervene appropriately while using the market [8]. The emissions trading system is the product of the combination of government and market, which is also a major innovation in environmental regulation. The 1990 amendments to the Clean Air Act first introduced the emission trading system (ETS) [9], which not only reduced U.S. emissions reductions from 1980 levels by a total of 40% but also provided new ideas for countries looking for effective ways to reduce SO_2_ emission reductions [10, 11]. Developed countries have borrowed from the U.S. approach to implement the ETS [12]. During improving air quality and safeguarding health [13], the potential of the ETS to promote SO_2_ emission reduction is gradually emerging [14].

Currently, Shi Wenming et al. (2021) show that China is the second-largest emitter of SO_2_, and finding more effective control methods has become a difficult task to promote SO_2_ emission reduction in China [15]. In the face of the harm caused by SO_2_ emissions, the Chinese government has repeatedly emphasized the promotion of SO_2_ emission reduction since 2006 and set SO_2_ emission reduction targets of 10%, 8%, and 15% in 2006, 2011, and 2016, respectively. During this period, in 2007, China launched ETS pilot projects in 11 provinces and cities in Jiangsu, Zhejiang, Tianjin, Hubei, Hunan, Chongqing, Hebei, Henan, Shanxi, Inner Mongolia, and Shaanxi, with the expectation of using market-based instruments to promote the sustainable and effective reduction of SO_2_ emissions. Jiang Lei et al. (2020) showed that central and provincial governments play a pivotal role in solving China’s environmental pollution problems and that governmental awareness of environmental protection contributes to significant reductions in SO_2_ emissions [16]. In this context, the effect of ETS implementation on SO_2_ emission reduction in China and the effective transmission pathway deserves further study. At the same time, ETS is an innovative environmental regulation tool. Finding the path to realize the effect of ETS on SO_2_ can help China find a practical path to manage SO_2_ and help production units establish the value of green and clean production, thus boosting sustainable development.

Therefore, based on the panel data of 285 Chinese cities (except Tibet) from 2004 to 2018, this paper investigates the effect of the ETS introduced in 2007 on SO_2_ emissions in China and the transmission mechanism using the difference-in-differences (DID) empirical approach and the mediating effect model.

The research of this paper is divided into six parts. The first part describes the reasons for the government to adopt environmental policies to inhibit SO_2_ and the current status of ETS implementation. The second part composes the current research results of scholars on the effect of ETS. The third part puts forward the research hypothesis of the effect of ETS on SO_2_ in China based on relevant theories and models. The fourth part establishes models required to test the transmission mechanism of the effect of ETS on China’s SO_2_ and lists the data sources. The fifth part uses the collated data to test the research hypothesizes and conduct analyses. The sixth part puts forward targeted policy recommendations on the specific challenges faced by China in promoting ETS at this stage.

The innovative points of this paper are as follows. First, the effect of ETS on SO_2_ is discussed at both national and regional levels by using the empirical method of DID. Second, city-level data are used for the analysis to make the results closer to the actual effect. Third, the transmission mechanism of the effect of ETS on SO_2_ is analyzed in terms of technological innovation and energy efficiency. Fourth, the robustness of the DID model is strengthened by the prior trend test, the placebo test, the exclusion of policy interference test, and the propensity score matching (PSM) test. It is worth mentioning that the research framework of this paper is not only applicable to Chinese ETS, but also to international ETS studies.

## 2 Literature review

As a typical model of market-based regulation, ETS has received much attention from international scholars in its creation and promotion. As the first country to implement ETS, Conrad Klaus and Kohn Robert (1996) studied that a market approach is more cost-effective in the United States than command and control regulations for pollution control [10], which has reduced U.S. SO_2_ emissions reductions by 40% from 1980 levels [17]. The ETS is ecologically efficient and economically effective; it plays a vital role in developing the future market for tradable permits [18]. However, there are some problems in ETS implementation, such as compliance costs are not being minimized, and the market is still not mature enough [19]. As the largest owner of the ETS, the effection of ETS implementation in E.U.has also received a high level of attention from scholars. During the implementation of the ETS, Swedish participation in the ETS has been enthusiastic. Still, in the absence of a sentiment market instrument that pays close attention to the pricing mechanism, it may severely negatively impact the system’s efficiency [20]. In addition, Rogge Karoline et al. (2012) show that because of the lack of rigor and predictability in the E.U. trading system, coupled with environmental constraints, the ETS may not provide sufficient incentives for fundamental changes in firms’ innovation activities at a level that ensures the achievement of long-term political goals [21]. Arto Iñaki et al. (2009) study points out that Spain, Austria, Italy, the U.K., and Sweden are better suited to the equalization scheme, which would greatly benefit the textile, non-metallic minerals, and paper industries, but would be particularly detrimental to the chemical, non-ferrous and other metals, and engineering industries [22].

Current research on ETS in China focuses on two main parts: the direct effect of ETS implementation and the transmission mechanism of ETS. Scholars have studied the feasibility of ETS implementation in China before its implementation and have noted that cap-and-trade offers the most cost-effective approach [23]. Shuangqing Xu and Bin Liu (2012) compared pollution control cases in the United States and China and pointed out that the command and control model can achieve efficient and cost effective results in the case of market failure and immature market environment [24].

Research studies on the direct effects of China’s ETS pilot can be divided into two main categories: economic effects and environmental effects. In terms of environmental benefits, in the early stages of ETS implementation, scholars questioned the effectiveness of the ETS in China. Zhen Lu (2011) pointed out that a national emissions trading scheme may not be suitable for China in the short term [25]. Zhengge Tu and Renjun Shen (2014) demonstrate that ETS fails to reduce the average pollution abatement cost based on the PSM-DID method [26]. Guang MingShi et al. (2016) use a game theory approach to show that pollution charging system (PCS) and ETS are not effective in motivating firms to reduce SO_2_ emissions [27]. As the policy advances, the research sample has extended in time and space, and inconsistencies in research findings emerge. At the regional level, some scholarly studies have pointed out that EST can not only effectively promote SO_2_ emission reduction [12], green development [28] in the pilot provinces, improving environmental efficiency [29] and raise environmental awareness in the surrounding areas [16]. On the contrary, Bingqing Hou et al. (2020) showed that ETS implementation hurts green development based on urban panel data [30]. At the firm level, Tang Maogang et al. (2021) showed that ETS has a significant contribution to SO_2_ emission reduction based on firm data [31], while Sousa Rita et al. (2020) showed that this contribution does not apply to resource-intensive industries [32]. In terms of economic benefits, the implementation of ETS can stimulate strategic behaviors such as technological innovation [33], production restructuring, and industrial restructuring [34], which not only improve the total factor productivity [35] but also increase the energy efficiency of the company [36]. Zhang H. and Fan LW. (2019) use provincial data to show that ETS is stimulating for provincial energy efficiency improvements, but not as effective as thought, and suggest that it is more appropriate to implement ETS in the industrial or transportation sectors than in the national sectors [37]. In addition, other scholars point out from the firm level that ETS still has difficulties in improving the productivity of firms [38] and the green total factor productivity of firms [30].

After confirming the existence of direct effects of ETS on the environment, scholars began to explore the transmission mechanism between ETS and the environment, which is the core area of concern in this paper. Scholars mainly analyze the transmission channels of ETS to the environment from two perspectives: internal ETS and external ETS. Gao Shuai and Wang Can. (2021) showed that the design of the ETS quota allocation on a uniform basis and allowing firms to bank the quota system can effectively encourage firms to participate in ETS and thus achieve environmental optimization [39]. Zhang Bing et al. (2010) show that transaction costs also reduce market efficiency, China’s coal price system is also a major factor affecting SO_2_ emissions trading market performance [40]. Ju Yiyi and Fujikawa Kiyoshi (2019) showed that through a cost transmission mechanism, the additional abatement costs caused by the national ETS drove an increase in household consumption in several energy-intensive industries [41]. Zhang Bing et al. (2013) showed that the number of cost savings and transactions was less than expected at the beginning of the project, and that enhanced policy interaction could effectively address this issue and thus contribute to SO_2_ reduction [42].

The study of extrinsic mechanisms. Zhang Jingxiao et al. (2021) showed that ETS can curb SO_2_ by increasing the efficiency of industrial green innovation, and that unbalanced economic and social growth could undermine any initial permit allocation scheme that might become a cornerstone of ETS [43]. Wang Lianfen et al. (2021) showed that the implementation of policies can promote the optimization of industrial structure through technological innovation, but this effect is influenced by economic development and the level of technology [44]. Zhang Shengling et al. (2021) showed that ETS achieves environmental optimization by increasing green total factor productivity (GTFP) and reducing investment in pollution-intensive industries in pilot provinces, confirming the existence of Porter’s hypothesis and the investment transfer transmission path [45]. Yang S. et al. (2021) showed that ETS promotes urban SO_2_ reduction by stimulating green technology innovation, promoting industrial restructuring, and directing investment toward green assets. Among them, the mediating effect of directing investment toward green assets is the strongest [46]. Because of the spatial spillover effect of the harm caused by the accelerated SO_2_ emissions [47], Du Gang et al. (2021) showed that ETS significantly stimulated green utility innovation in and around the pilot areas and that there was a masking effect of government intervention in the process [48].

In general, the shortcomings of the current research results are as follows. Firstly, the discussion of ETS is controversial and lacks heterogeneity at the regional level. Second, most scholars study policy effects at the provincial level, which can make policy effect estimates biased. Third, scholars have studied the direct effect of ETS on environmental optimization, but less attention has been paid to the transmission mechanism between ETS and environmental effects. Fourth, the existing literature is also crude in terms of data interpretation and model robustness tests.

## 3 Research hypothesis

### 3.1 The effect of ETS on SO_2_

The ETS, which combines market mechanisms with government regulation, is a significant innovation in environmental law. The core idea is that by setting a maximum amount of pollution emissions in a region and allocate a share of the emissions to each enterprise [49], which is directly linked to the production costs of enterprises. Firms that emit less than the allotted amount can sell their excess share in the emissions trading market and receive a profit, reducing the production costs of low-polluting firms. In contrast, enterprises whose emissions are higher than the allocated amount need to buy the corresponding share in the emissions trading market, which raises the production cost of high polluting enterprises to some extent. The cost-benefit theory holds that financial decisions should be based on the principle that the benefits outweigh the costs, and this decision is feasible only when the input costs are less than the benefits brought by the output. At this time, enterprises with strong pollution control ability will adjust their production mode more actively because of the benefits to achieve a sustainable and effective reduction of emissions. The firms with weak emission capacity will also adapt their production mode because of the increased cost to reduce their negative externalities on the environment. As SO_2_ is the main component of pollutants in China, hypothesis H1 is proposed in this paper.

H1: ETS directly affects SO_2_ and can significantly suppress SO_2_.

### 3.2 Path of government intervention (GOV)

Because the environment is a public good characterized by externalities and property rights are difficult to define clearly, environmental issues require a joint role of government and markets [50]. Traditional environmental regulation tends to be dominated by administrative forces, i.e., local governments tend to use the command-and-control environmental scheme to restrict corporate emissions behavior. This regulation creates barriers to inter-regional factor flows and intensifies market fragmentation, harming eco-efficiency [51]. At the same time, the trade-off between governance costs and rent-seeking costs can lead to low awareness of cleaner production and insufficient incentives for energy conservation and emission reduction among enterprises [52]. In addition, information asymmetry often leads to the failure of command-and-control environmental regulation [53]. Unlike traditional environmental schemes, ETS, as a market-based one, cedes part of its power to the market by establishing an emissions trading market, which fully utilizes the decisive role of the market in resource allocation and enhances the speed of elimination of backward production capacity. In addition, the revenue gained by the enterprises with strong pollution control ability in the trading process also plays a particular incentive effect. Thus this paper proposes hypothesis H2.

H2: ETS indirectly affects SO_2_ and can suppress SO_2_ by reducing GOV.

### 3.3 Path of green technology innovation (GTI)

Based on Porter’s hypothesis (1995), appropriate environmental regulation can lead to more innovative activities by firms, which will increase their productivity [54]. Thus offsetting the costs of environmental protection and improving their profitability in the market and product quality may give domestic firms a competitive advantage in the international market and, at the same time, may increase industrial productivity. ETS has changed the concept from emissions are punishable to profitable as a new environmental regulation tool, which puts pressure on production units to discharge emissions and warns them that high value-added, low pollution, and green sustainability are the future direction of economic growth development. This approach motivates production units to engage in GTI actively to achieve effective long-term reductions in pollutant emissions and thus meet the reduction targets of environmental policies. Therefore, this paper proposes hypothesis H3.

H3: ETS indirectly affects SO_2_ and can suppress our SO_2_ through GTI.

### 3.4 Path of resource utilization efficiency (RUE)

The implementation of ETS is based on production units not only in terms of emission pressure but also in terms of intensified market competition. And market competition is characterized by the flow of factors from inefficient sectors to efficient sectors, thus achieving last place elimination, which often shows a positive correlation with this firm productivity [55]. Therefore, firms should reduce their pollution emissions and improve the efficiency of resource use to prevent being eliminated in the process of market competition. Under a perfectly competitive market, and emission shares can be traded according to firm demand. In reality, however, Tombe and Winter (2015), in their study, combined environmental regulation with resource allocation for the first time, point out that environmental regulation set based on pollution intensity carries significant information asymmetry [56]. This asymmetry may lead to resource misallocation. As a result, there is a possibility that a few firms may purchase and store emission rights far over their emission quotas to gain monopoly revenues or for future use, with consequences such as a decrease in product market production [57], which results in higher compliance costs for buyers of emission shares. To be able to thrive under the pressure of environmental regulation, production units will increase the efficiency of resource use to reduce production costs. Therefore, this paper proposes hypothesis H4.

H4: ETS indirectly affects our SO_2_ and can suppress SO_2_ by enhancing RUE.

**Fig 1:**
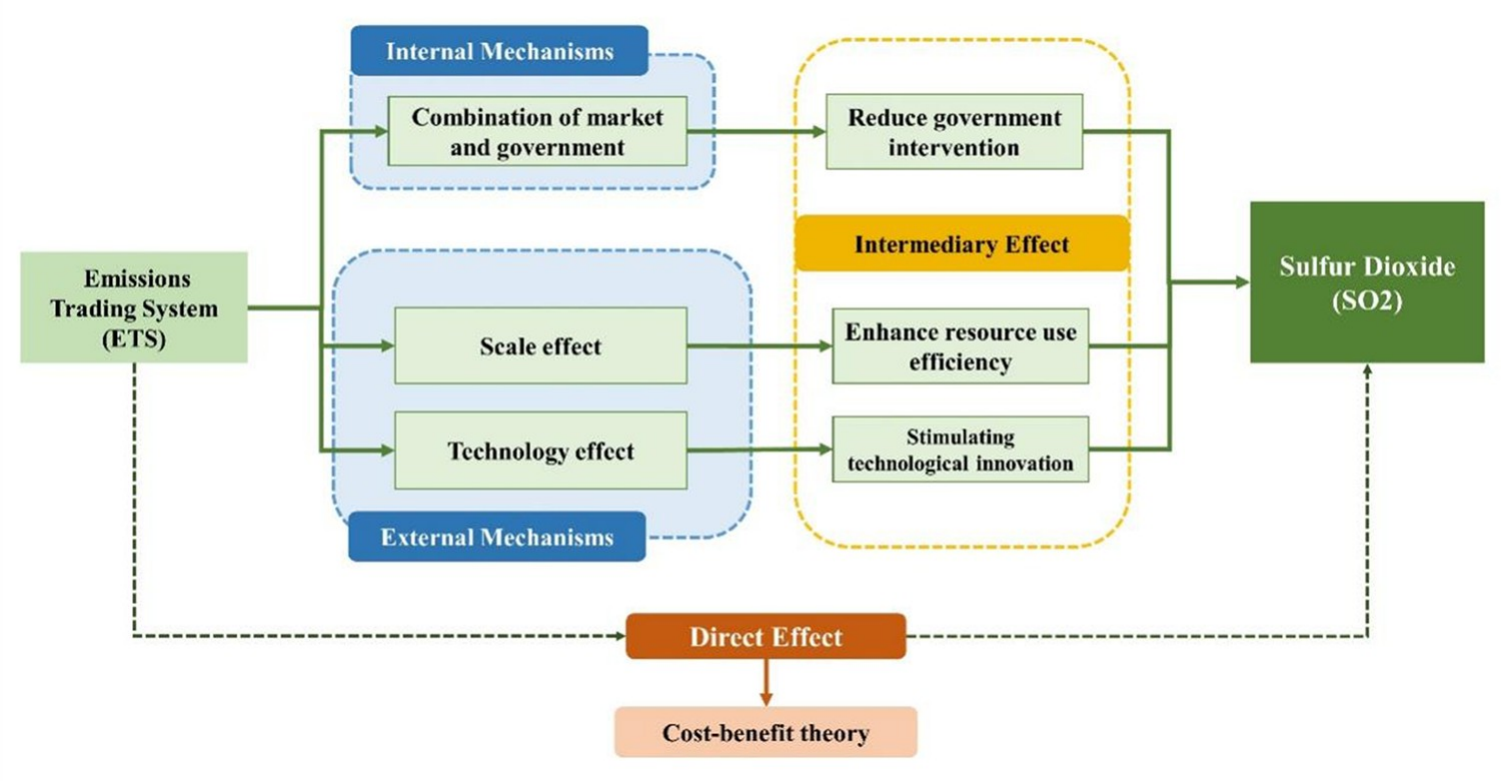
Research structure diagram.

Based on the above theoretical analysis, this paper studies the transmission mechanism of the effect of ETS on SO_2_ in China from the perspective of GOV, GTI, and RUE, and the structure of the study is shown in Fig 1.

## 4 Methodology and data

### 4.1 Model setting

#### 4.1.1 Basic model

Most of the existing literature uses the difference in differences method for the assessment of policy effects. The sample is divided into treatment and control groups and combined with a fixed-effects model, and the data are then compared between the two periods. This approach yields relatively accurate results for assessing policy effects, largely avoids endogeneity problems, and omits variable bias. For the study of this paper, a difference in differences model is developed, as shown in Eq (1).


$${{SO}_{2}}_{it}=\alpha_{0}+\alpha_{1}{Policy}_{it}\times {Year}_{t}+\alpha_{2}{Control}_{it}+\mu_{i}+\lambda_{t}+\varepsilon_{it}$$ (1)

**Fig 2:**
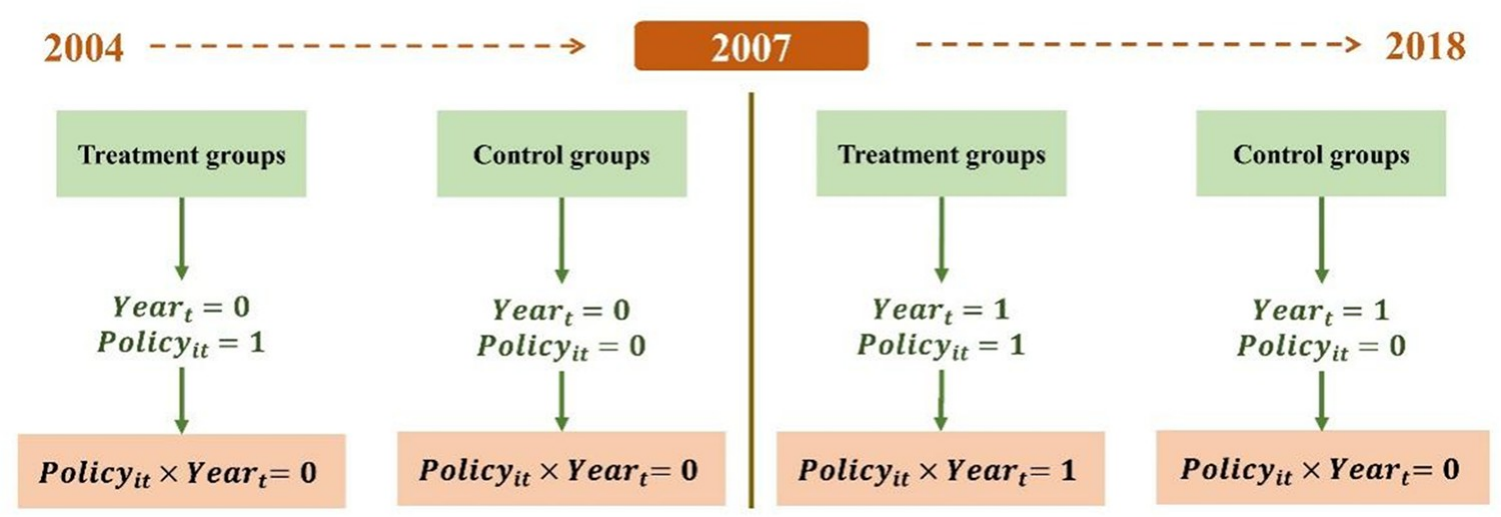
Policy dummy variable description.

In Eq (1), the explanatory variable ${{SO}_{2}}_{it}$ is sulfur dioxide emissions, *Policy*_*it*_ and *Year*_*t*_ are both 0–1 dummy variables reflecting the regional policy implementation, *i* is the city, and *t* is the time. Based on the government work report, 2007 was taken as the policy implementation year, and the values of treatment group and control group are shown in Fig 2. *Control*_*it*_ is a series of control variables, *μ*_*i*_ and *λ*_*t*_ are individual fixed effects and time fixed effects, and *ε*_*it*_ is random error influenced by the province time trend term.

### 4.1.2 Parallel trend test model

In assessing policy effects by the DID method, the common trend is a crucial assumption that largely determines the accuracy of the model assessment. Therefore, in this paper, two-period and multi-period parallel trend tests are conducted in the study to analyze the effect of ETS on SO_2_ emissions, and a multi-period parallel trend test model is established as shown in Eq (2).


$${{SO}_{2}}_{it}=\beta_{0}+\sum_{t=2004}^{2018}\beta_{1}{Policy}_{it}\times d_{t}+\beta_{2}{Control}_{it}+\mu_{i}+\lambda_{t}+\varepsilon_{it}$$ (2)

In Eq (2), *d*_*t*_ is a dummy variable of time, if *t*>2007,then *Policy*_*it*_×*d*_*t*_ = 1, if *t*≤2007, then *Policy*_*it*_×*d*_*t*_ = 0. *β*_1_ is the coefficient of the cross product term, which is the average treatment effect. Theoretically, the policy effect is significant if the average treatment effect is not significant before the policy is implemented, but the average treatment effect is significant after the policy is implemented.

#### 4.1.3 Intermediary effect model

Based on the hypotheses in section 3, this paper analyzes the effect of ETS on SO_2_ from two perspectives: GOV, GTI, and RUE, based on the mediating effect model test proposed by Baron and Kenny’s (1986) [58]. M is used to represent all mediating variables involved in this paper. The mediating effect test model is shown in Eqs (3)–(5).


$${{SO}_{2}}_{it}=\alpha_{0}+\alpha_{1}{Policy}_{it}\times {Year}_{t}+\alpha_{2}{Control}_{it}+\mu_{i}+\lambda_{t}+\varepsilon_{it}$$ (3)

$$M_{it}=\beta_{0}+\beta_{1}{Policy}_{it}\times {Year}_{t}+\beta_{2}{Control}_{it}+\mu_{i}+\lambda_{t}+\varepsilon_{it}$$ (4)

$${{SO}_{2}}_{it}=\gamma_{0}+\gamma_{1}{Policy}_{it}\times {Year}_{t}+\gamma_{2}M_{it}+\gamma_{3}{Control}_{it}+\mu_{i}+\lambda_{t}+\varepsilon_{it}$$ (5)

The test steps are as follows. In the first step, test whether the interaction term coefficient *α*_1_ in Eq (3) is significant for the second step of the test. If not significant, the causal effect is not apparent to abandon the intermediary effect test. The second step tests whether the *β*_1_ in Eq (4). If significant, continue the third step of the intermediary effect test. On the contrary, then the intermediary effect is not significant, the test is terminated. The third step tests whether *γ*_1_ and *γ*_2_ in Eq (5) is significant, if only *γ*_1_ is significant, it is a complete mediation effect. If both *γ*_1_ and *γ*_2_ are significant, it is a partial mediation effect. In addition, if *β*_1_×*γ*_2_ and *γ*_1_ have the same sign, then the argument is based on the mediating effect, and if *β*_1_×*γ*_2_ and *γ*_1_ have different signs, then the argument is based on the masking effect.

### 4.2 Data selection

Explained variables. In order to investigate the impact of ETS on China’s SO_2_ emissions, this paper selects SO_2_ emissions in various regions as an explained variables. The data source is China City Statistical Yearbook and the data description is shown in Table 1. The acronyms involved in the study of this paper are shown in Table 2.

**Table 1:** Variable description.

Description	Unit	Obs	Mean	Std. Dev.	Min	Max
Sulfur dioxide emissions	—	4,275	5.99	8.46	0.00	152.63
Gross Domestic Product	—	4,275	1437.195	2015.286	35.8573	24050.48
Tertiary Industry Value Added/ Gross Domestic Product	%	4,275	38.35	9.73	8.58	85.34
Total population at the end of the year	—	4,275	443.51	396.18	16.76	11098.40
Gross Domestic Product /Total electricity consumption	—	4,275	9.00	18.29	0.00	293.92
Per capita income	—	4,275	35066.28	19262.29	6207.11	135815.60
Number of cell phones	—	4,275	344.53	426.84	4.74	6460.21
Science and technology expenditure/government expenditure	—	4,275	18.25	5.52	1.04	43.49
Green Invention-based Patent Applications	—	4,275	211.18	872.45	0.00	19674.00
Green Utility Patent Applications	—	4,275	188.75	595.41	0.00	9506.00
Gross Domestic Product /energy consumption	—	4,275	1.19	0.95	0.02	6.82
Calculated by MaxDEA	—	4,275	1.07	0.35	0.01	4.44
government expenditure / Gross Domestic Product	%	4,275	0.18	0.12	0.01	0.92

Data source: China Statistical Yearbook, Environment Statistical Yearbook, Energy Statistical Yearbook, National Patent Statistics Office official website

**Table 2:** Abbreviations table.

Name	Description
**SO**_**2**_	Sulfur dioxid
**ETS**	Emission trading policy
**GOV**	Government intervention
**GTI**	Green technology innovation
**GITA**	Invention-based patent innovation level
**GITB**	Utility-based Patent Innovation Level
**RUE**	Resource Utilization
**ECS**	Energy use level
**TFP**	Total Factor Productivity

Control variables. In order to avoid the influence of omitted variables, this paper adopts the following control variables. (1) Economic development (economy): Grossman and Krueger (1995) argue that regional economic growth may be accompanied by excessive consumption of environmental resources [59, 60], exacerbating environmental degradation [61]. But the willingness to use environmental resources in exchange for economic growth gradually declines when economic development reaches a certain level. (2) Per capita income (income): The environmental Kuznets curve states an inverted U-shaped relationship between per capita income level and ecological pollution, and SO_2_ can generally satisfy the environmental Kuznets curve [62]. (3) Industrial structure (structure): As an essential link between human economic activities and air quality, changes in industrial structure can have an impact on the environment [63]. For example, a decrease in the share of secondary industry output in GDP can significantly reduce SO_2_ pollution [63]. (4) Population size (population): According to Dietz T. and Rosa E. (1997), the environmental impact of population growth is characterized by significant diseconomies of scale [64]. On the one hand, population expansion brings direct environmental pressure, increasing the consumption of exhaustible resources and leading to environmental degradation. On the other hand, population growth intensifies the demand for industrial products, and the expansion of industrial production brings higher levels of pollutant emissions. (5) Electricity efficiency (electricity): As the economy grows and the scale of production increases, the increase in electricity efficiency can impact SO_2_ [19]. (6) Infrastructure (infrastructure): With economic development, urban infrastructure has led to more active production and life, which has a certain impact on the environment [65]. (7) Technology investment (technology). Government spending on technology can help finance technology research and development units and contribute to the development of science and technology, which is led by the value of green development and tends to favor environmentally beneficial technology development, thus optimizing environmental quality [66]. Among them, GDP is deflated with 2003 as the base period. The above indicators are calculated from the China Statistical Yearbook. Before processing, the raw data of the above indicators are obtained from China Statistical Yearbook, and the data description is shown in Table 1.

Mediating variables. This paper analyzes the effect of ETS on SO_2_ from three perspectives: GOV, GTI, and RUE.

In terms of the perspective of GOV, local governments can direct the flow of resources through investment and fiscal spending, impacting production and thus the environmental quality. Fiscal is an effective tool for the government to address resource allocation. Therefore, this paper draws on Du Gang et al.’s (2021) study using the ratio of government fiscal expenditure to GDP for the year to measure the level of government intervention [48].

GTI perspective. Since there is a specific time distance from the application to the acquisition of green patents, some of the green patents obtained in the year of policy implementation were applied for before the performance of the policy, and policy factors less influence this part of the patent application. In addition, the patent acquisition is influenced by multiple factors. Therefore, this paper selects the number of GTIA and the number of GTIB applications to measure green technology innovation. The data source is the National Bureau of Statistics.

RUE perspective. This paper analyzes the mediating effect of resource use efficiency from two perspectives: ECS and TFP. First is the energy efficiency perspective. In this paper, the ratio between GDP and energy consumption is used as an indicator to measure energy efficiency, where GDP data are obtained from the China Urban Statistical Yearbook and deflated with 2003 as the base period. Energy consumption is estimated using nighttime lighting data. Numerous scholars have demonstrated a correlation between the number of lights and energy consumption [67–70]. The basic logic is that higher light levels at night indicate more economic activity at night, implying a higher level of economic development and corresponding energy consumption. Chinese scholar Wu Jiansheng et al. (2009) demonstrated the relationship between nighttime lighting and energy consumption in exponential, linear, and logarithmic relationships in China, with the most robust linear relationship [71]. Xiao Hongwei (2018) showed that the accuracy of this estimation method is as high as 99% [72]. Therefore, this paper uses the linear correlation between energy consumption and total nighttime lights in 30 provinces of China to establish a linear simulation model of energy consumption in China by regions through regression analysis. Considering the accuracy problem of downscaling model inversion, the linear model without intercept is adopted in this paper, and its formula is shown in (6).


$$E_{it}=k_{t}{DN}_{it}$$ (6)

In Eq (6), *E*_*it*_ is the statistical value of energy consumption in province *i* in year *t*; *k*_*t*_ is the coefficient in year *t*; *DN*_*it*_ is the sum of grayscale values of all rasters in province *i* in year *t*.

Second is the total factor productivity perspective. Total factor productivity (TFP) measurement methods can be broadly classified into the stochastic frontier analysis (SFA) and data envelopment analysis (DEA). Compared with the overly idealized expressive relationships among variables, DEA only derives the optimal weights from the actual input-output data of decision units, which has strong objectivity [73]. Therefore, this paper uses capital, labor, and energy as inputs and GDP as output to estimate the allocation effect. The capital data are estimated using the perpetual inventory method. Meanwhile, labor data are the average number of employees per year, while energy is the total amount of energy input per year. The data source is the China City Statistical Yearbook. Among these variables, those affected by price factors are deflated using 2003 as the base period.

## 5 Results and discussion

### 5.1 Fundamental analysis

**Fig 3:**
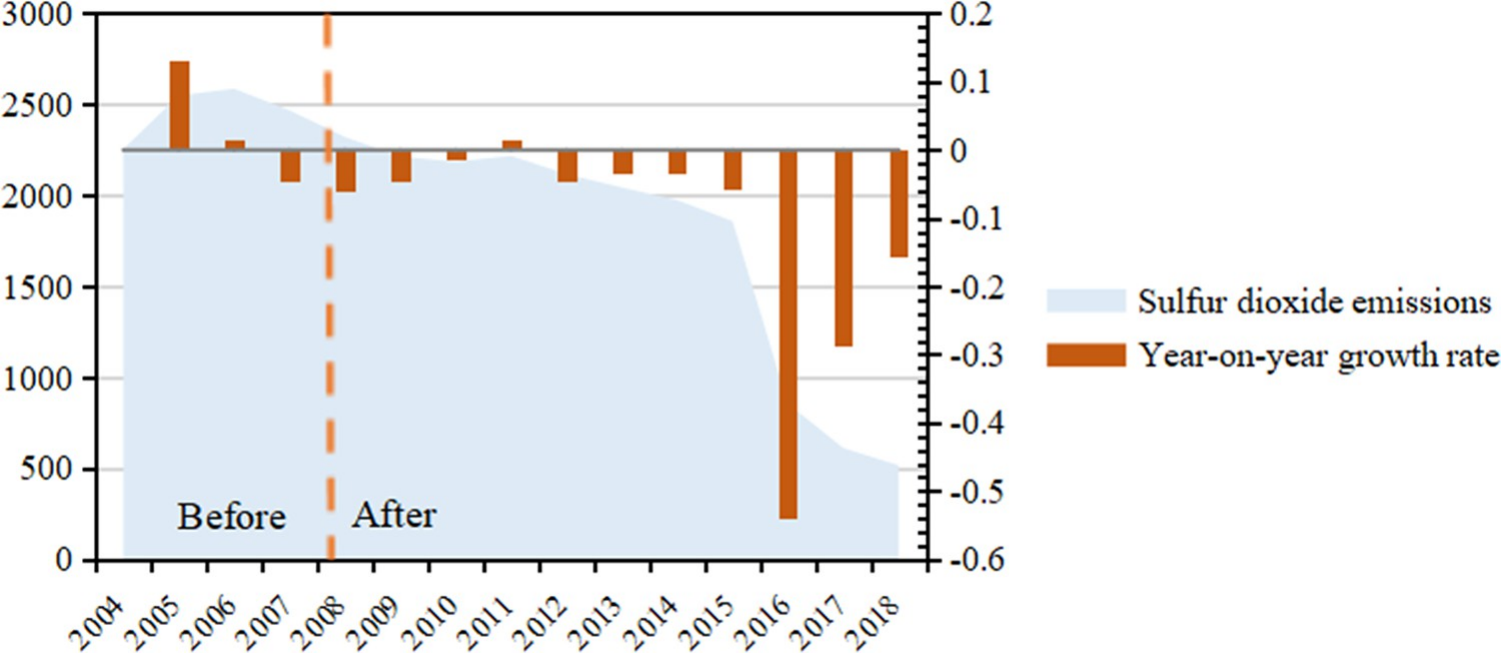
Sulfur dioxide emission request from 2004–2018.

Before the logarithmic treatment of SO_2_ emissions, we conducted a fundamental analysis of SO_2_ emissions, as shown in Fig 3. From Fig 3, before the implementation of ETS in 2007, the overall Sulphur dioxide emissions in China increased, while Sulphur dioxide emissions showed a decreasing trend after 2007. Besides, the growth rate of Sulphur dioxide emissions also changed from positive to negative, which shows that China has effectively reduced Sulphur dioxide emissions during this period. However, the effect of ETS as an environmental regulation policy tool generated in this period cannot be judged directly.

### 5.2 Baseline model test results

Before conducting the baseline regression, this paper used the correlation coefficient analysis to analyze the control variables for multicollinearity. As can be seen from the Table 3, the variance inflation factors (VIFs) are below 10, and the tolerances are above 0.1, so there is no multicollinearity in this paper as far as the control variables are concerned [74, 75]. In addition, the variables in this paper were subjected to the Hausman test, which significantly rejected the original hypothesis, so in the baseline regression, this paper selected the DID model with double fixation of time and location to assess the effect of ETS on SO_2_.

**Table 3:** Variable tests.

Variable	VIF	1/VIF
**Infrastructure**	6.62	0.151134
**Economy**	5.89	0.169777
**Population**	3.06	0.326985
**Income**	2.95	0.338712
**Electricity**	1.90	0.527508
**Structure**	1.32	0.757612
**Technology**	1.12	0.893953
**Mean VIF**	3.26	
**Hausman**	197.84***	

Standard errors in parentheses

*** p<0.01

** p<0.05

* p<0.1

For the study of the effect of ETS on SO_2_, this paper adopts the stepwise regression method to present the effect of ETS on SO_2_ in China, and the baseline regression results are shown in Table 4. From Table 4, the effect of ETS on SO_2_ is significant, and compared with the control group, the SO_2_ emission of the treatment group is reduced by 26.44%. Thus, hypothesis H_1_ that ETS directly affects SO_2_ emissions and can effectively suppress SO_2_ emissions in China is confirmed.

**Table 4:** Baseline regression results.

	(1)	(2)	(3)	(4)	(5)
VARIABLES	SO_2_	SO_2_	SO_2_	SO_2_	SO_2_
**ETS**	-0.2728***	-0.2675***	-0.2642***	-0.2661***	-0.2644***
	(0.0437)	(0.0438)	(0.0435)	(0.0434)	(0.0432)
**Economy**		-0.0180	0.1563	0.6609***	0.6549***
		(0.1286)	(0.1297)	(0.2354)	(0.2340)
**Structure**		-0.0088***	-0.0097***	-0.0096***	-0.0092***
		(0.0024)	(0.0023)	(0.0023)	(0.0023)
**Population**			-0.0816	-0.0806	-0.0763
			(0.0559)	(0.0559)	(0.0556)
**Electricity**			-0.1010***	-0.0989***	-0.0944***
			(0.0135)	(0.0135)	(0.0135)
**Income**				-0.5961**	-0.6105***
				(0.2321)	(0.2308)
**Infrastructure**					0.2182***
					(0.0333)
**Technology**					-0.0045**
					(0.0021)
**Constant**	10.4402***	10.8497***	10.3384***	13.0225***	12.2918***
	(0.0329)	(0.7871)	(0.8430)	(1.3421)	(1.3406)
**Year**	Yes	Yes	Yes	Yes	Yes
**Province**	Yes	Yes	Yes	Yes	Yes
**Observations**	4,275	4,275	4,275	4,275	4,275
**R-squared**	0.5026	0.5045	0.5117	0.5125	0.5183

Standard errors in parentheses

*** p<0.01

** p<0.05

* p<0.1

### 5.3 Parallel trend test results

**Fig 4:**
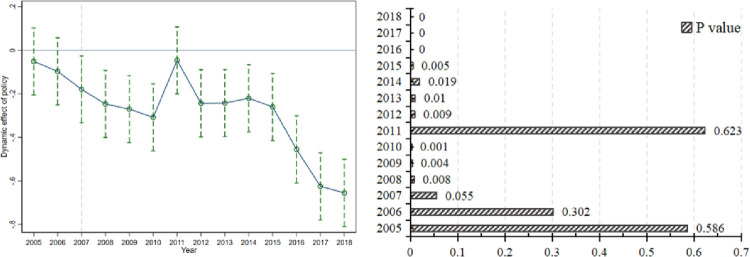
Multi-period parallel trend test.

Considering that the implementation of the ETS pilot is a continuous dynamic adjustment process, it is necessary to consider further the dynamic marginal effect of ETS on SO_2_ emission reduction in China. In the test process, 2004 is taken as the base period of policy implementation, and the test results are shown in Fig 4. From Fig 4, overall, the coefficient of the interaction term changes from positive to negative after the implementation of the policy. After implementing ETS in 2007, ETS significantly suppressed SO_2_ emissions at a 90% confidence level, especially after 2012, when the confidence level stabilized above 95%. To explain the insignificance in 2011, the government announced a 2011 plan for another market-based environmental regulation policy, namely the carbon emissions trading policy. This policy adds additional pressure to reduce carbon emissions and distracts the production units, which the effect of ETS is somewhat disturbed.

### 5.4 Analysis of ETS transmission mechanism

#### 5.4.1 GOV conduction pathway

Based on the theoretical hypothesis proposed in section 3, the intermediary effect of GOV is tested in this paper, and the test results are shown in Table 5. From the test results in Table 5, we can see that the mediating effect of GOV does exist in the process of SO_2_ reduction by ETS, and ETS can suppress SO_2_ by reducing GOV, which also confirms the validity of hypothesis H_2_.

**Table 5:** Results of GOV mediating effect test.

	(1)	(2)	(3)
VARIABLES	SO_2_	GOV	SO_2_
**EST**	-0.2644***	-0.0217***	-0.2529***
	(0.0432)	(0.0050)	(0.0432)
**GOV**			0.5342***
			(0.1379)
**Economy**	0.6549***	-0.1224***	0.7208***
	(0.2340)	(0.0269)	(0.2340)
**Structure**	-0.0092***	-0.0001	-0.0088***
	(0.0023)	(0.0003)	(0.0023)
**Population**	-0.0763	-0.0100	-0.0671
	(0.0556)	(0.0064)	(0.0554)
**Electricity**	-0.0944***	-0.0042***	-0.0914***
	(0.0135)	(0.0015)	(0.0135)
**Income**	-0.6105***	-0.0059	-0.6119***
	(0.2308)	(0.0265)	(0.2301)
**Infrastructure**	0.2182***	0.0584***	0.1842***
	(0.0333)	(0.0038)	(0.0342)
**Technology**	-0.0045**	-0.0036***	-0.0076***
	(0.0021)	(0.0003)	(0.0027)
**Constant**	12.2918***	0.6925***	12.0188***
	(1.3406)	(0.1539)	(1.3406)
**Year**	Yes	Yes	Yes
**Province**	Yes	Yes	Yes
**Observations**	4,275	4,275	4,275
**R-squared**	0.5183	0.6088	0.5212

Standard errors in parentheses

*** p<0.01

** p<0.05

* p<0.1

This paper gives the following explanation for this phenomenon. As a market-based incentive-based environmental regulation tool, ETS makes it possible to build a platform for enterprises to trade emission shares by establishing an emission rights trading market. On the one hand, the seller of the share of pollution gains revenue in trading and then invests the earned income into production, which stimulates the seller’s incentive to treat pollution and relieves the pressure of government subsidies. The government can reduce its investment accordingly. On the other hand, for the share buyer, the addition of a market mechanism enhances the production cost of the share buyer. It enhances the buyer’s awareness of self-reduction under market competition. Therefore, the government will simplify and decentralize the government, give up more power to the market for allocation and regulation, and enhance the awareness and ability of enterprises to treat pollution through market competition in all aspects. Thus effectively reducing pollution emissions and achieving environmental optimization.

#### 5.4.2 GTI conduction pathway

Based on the Porter hypothesis theory proposed in section 3, this paper tests the small and medium effects of GTI. Since GTI is composed of GTIA and GTIB, this paper tests the mediation path of GTI from these two perspectives, and the test results are shown in Table 6. Among them, Table 6-(2) and Table 6-(3) show the test results of GTIA, and Table 6-(4) and Table 6-(5) show the test results of GTIB. From Table 6, it is clear that the mediating effect of GTIA does exist in the process of achieving SO_2_ emission reduction by ETS, while GTIB has a masking impact on the process.

**Table 6:** Results of GTI mediating effect test.

	(1)	(2)	(3)	(4)	(5)
VARIABLES	SO_2_	GTIA	SO_2_	GTIB	SO_2_
**ETS**	-0.2644***	0.1130**	-0.2612***	0.1215***	-0.2690***
	(0.0432)	(0.0458)	(0.0432)	(0.0388)	(0.0432)
**GTIA**			-0.0287*		
			(0.0150)		
**GTIB**					0.0375**
					(0.0177)
**Economy**	0.6549***	-0.0797	0.6526***	0.1570	0.6490***
	(0.2340)	(0.2482)	(0.2339)	(0.2100)	(0.2339)
**Structure**	-0.0092***	-0.0026	-0.0093***	0.0015	-0.0093***
	(0.0023)	(0.0025)	(0.0023)	(0.0021)	(0.0023)
**Population**	-0.0763	0.1192**	-0.0728	0.1593***	-0.0822
	(0.0556)	(0.0589)	(0.0556)	(0.0499)	(0.0556)
**Electricity**	-0.0944***	-0.0065	-0.0945***	-0.0710***	-0.0917***
	(0.0135)	(0.0143)	(0.0135)	(0.0121)	(0.0136)
**Income**	-0.6105***	0.8864***	-0.5850**	0.8256***	-0.6414***
	(0.2308)	(0.2447)	(0.2311)	(0.2071)	(0.2311)
**Infrastructure**	0.2182***	0.0208	0.2188***	0.0044	0.2180***
	(0.0333)	(0.0353)	(0.0333)	(0.0299)	(0.0333)
**Technology**	-0.0045**	0.0014	-0.0045**	0.0008	-0.0045**
	(0.0021)	(0.0023)	(0.0021)	(0.0019)	(0.0021)
**Constant**	12.2918***	-7.3799***	12.0801***	-7.9566***	12.5903***
	(1.3406)	(1.4218)	(1.3447)	(1.2032)	(1.3474)
**Year**	Yes	Yes	Yes	Yes	Yes
**Province**	Yes	Yes	Yes	Yes	Yes
**Observations**	4,275	4,275	4,275	4,275	4,275
**R-squared**	0.5183	0.8103	0.5188	0.8362	0.5189

Standard errors in parentheses

*** p<0.01

** p<0.05

* p<0.1

The interpretation of this result is as follows. In ETS implementation, production units need to purchase emission credits to meet their emission demand, which makes the emission volume directly related to the production cost of production units. Under the dual pressure of environmental regulation and industrial development, production units inevitably have to find a balance between the two. At the same time, since the research process of GTI requires a large amount of capital and is led by the value of low pollution and low emission, the money is more inclined to the units with clean production. Therefore, production units will continue to increase GTI. Although GTIB, which takes less time, consumes fewer resources, and is highly practical, may be more preferred. However, a utility model patent is mainly a practical new technical solution for a product’s shape, structure, or combination, not technological innovation that transforms the production method. Therefore, utility model patent innovations that follow previous production methods do not significantly suppress SO_2_. In contrast, GTIA, as a green innovation in production methods, can effectively curb the development of ETS, although it is complex and has a long cycle time. Production units will also focus on increasing the investment in GTIA for long-term development, thus achieving a change in production methods. In addition, it cannot be excluded that the inconsistency of the research and application cycle between GTIA and GTIB makes the stimulating effect of ETS on GTIB more evident than that of GTIA.

#### 5.4.3 RUE conduction pathway

In order to investigate whether ETS can achieve SO_2_ emission reduction through RUE. This paper measures RUE through two indicators, ECS and TFP, where ECS is energy use efficiency and TFP is total factor productivity, and the test results are shown in Table 7. Table 7 shows that ETS can suppress the emission of SO_2_ through ECS and TFP. Thus, the hypothesis H_4_ " ETS can curb SO_2_ emissions in China by improving the efficiency of resource utilization." is confirmed.

**Table 7:** Results of RUE mediating effect test.

	(1)	(2)	(3)	(4)	(5)
VARIABLES	SO_2_	ECS	SO_2_	SO_2_	SO_2_
**EST**	-0.2644***	0.0658***	-0.2517***	0.0383**	-0.2605***
	(0.0432)	(0.0088)	(0.0435)	(0.0195)	(0.0432)
**ECS**			-0.1935**		
			(0.0783)		
**TFP**					-0.1036***
					(0.0352)
**Economy**	0.6549***	0.7841***	0.8066***	0.0202	0.6569***
	(0.2340)	(0.0474)	(0.2418)	(0.1055)	(0.2338)
**Structure**	-0.0092***	-0.0008*	-0.0094***	-0.0021**	-0.0095***
	(0.0023)	(0.0005)	(0.0023)	(0.0011)	(0.0023)
**Population**	-0.0763	0.0182	-0.0727	0.0133	-0.0749
	(0.0556)	(0.0113)	(0.0555)	(0.0250)	(0.0555)
**Electricity**	-0.0944***	0.0307***	-0.0884***	0.0734***	-0.0868***
	(0.0135)	(0.0027)	(0.0137)	(0.0061)	(0.0137)
**Income**	-0.6105***	0.0551	-0.5998***	-0.1625	-0.6273***
	(0.2308)	(0.0468)	(0.2306)	(0.1040)	(0.2306)
**Infrastructure**	0.2182***	0.0028	0.2188***	-0.0726***	0.2107***
	(0.0333)	(0.0067)	(0.0333)	(0.0150)	(0.0334)
**Technology**	-0.0045**	0.0005	-0.0044**	0.0017*	-0.0043**
	(0.0021)	(0.0004)	(0.0021)	(0.0010)	(0.0021)
**Constant**	12.2918***	-5.6703***	11.1945***	2.6481***	12.5662***
	(1.3406)	(0.2717)	(1.4114)	(0.6044)	(1.3426)
**Year**	Yes	Yes	Yes	Yes	Yes
**Province**	Yes	Yes	Yes	Yes	Yes
**Observations**	4,275	4,275	4,275	4,275	4,275
**R-squared**	0.5183	0.8026	0.5191	0.1504	0.5194

Standard errors in parentheses

*** p<0.01

** p<0.05

* p<0.1

In response to this empirical result, this paper tries to make the following explanation. ETS is implemented by setting a maximum amount of pollution emissions in a region and assigning the right to discharge emissions to each production unit. Unlike traditional environmental regulation, ETS combines government regulation with market mechanisms to create a market for emissions trading among producers. Production units can buy or sell their share of emissions in the emissions trading market through their production. The market mechanism often leads to a reallocation of factors, driving the flow of factors from inefficient to efficient sectors [55]. Moreover, information asymmetries may lead to distortions in factor allocation and thus affect production [57]. Therefore, during the ETS implementation process, production units will add energy source efficiency to reduce SO_2_ emissions while ensuring production. At the same time, enterprises will also carry out internal business model innovation, more efficient use of all factors to improve the overall resource utilization efficiency. This behavior can not only promote enterprises in the market competition to have the strength to obtain more resources but also enable enterprises to obtain more revenue in the emission trading market to form a virtuous circle.

### 5.5 Robustness tests

#### 5.5.1 Propensity score matching test

**Fig 5:**
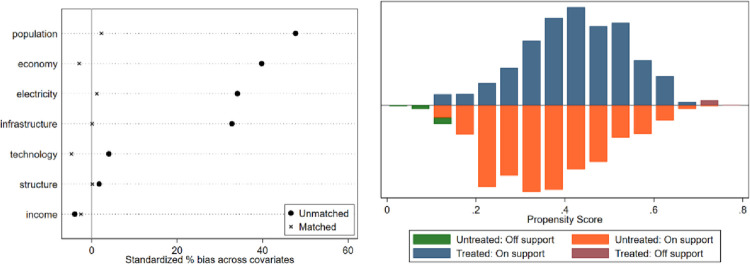
Equilibrium test for nearest neighbor matching and propensity score (PS) plot.

**Fig 6:**
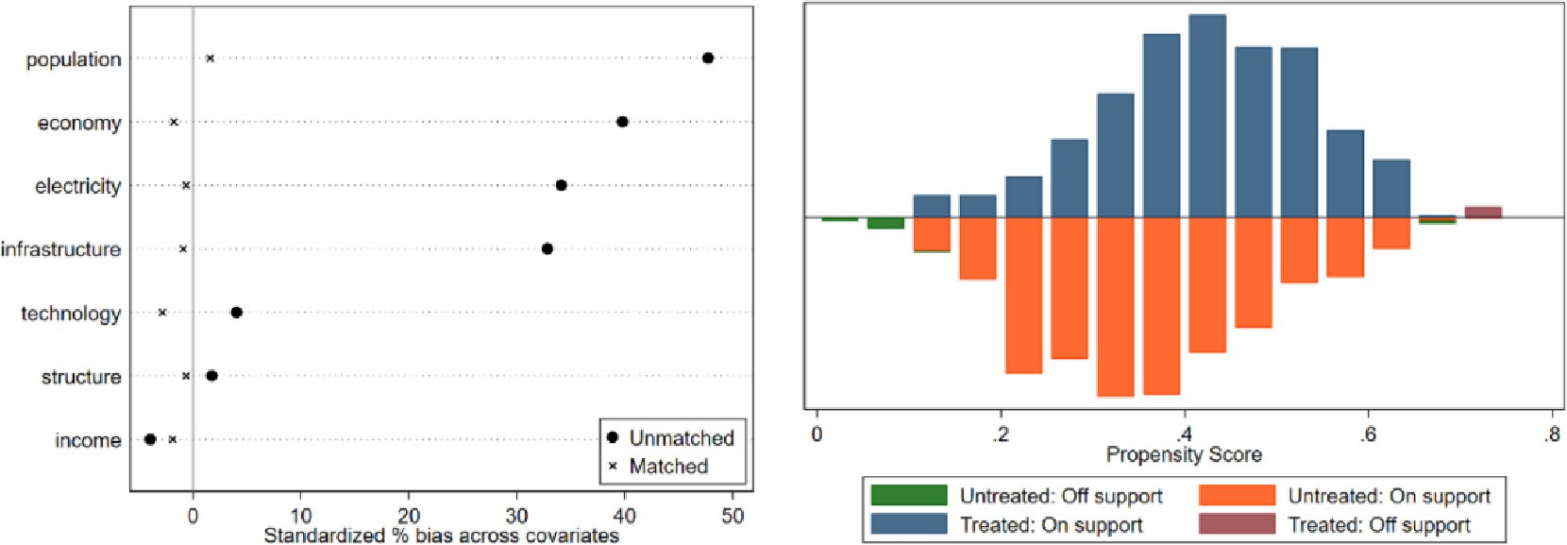
Radius matching equilibrium test and PS plot.

**Fig 7:**
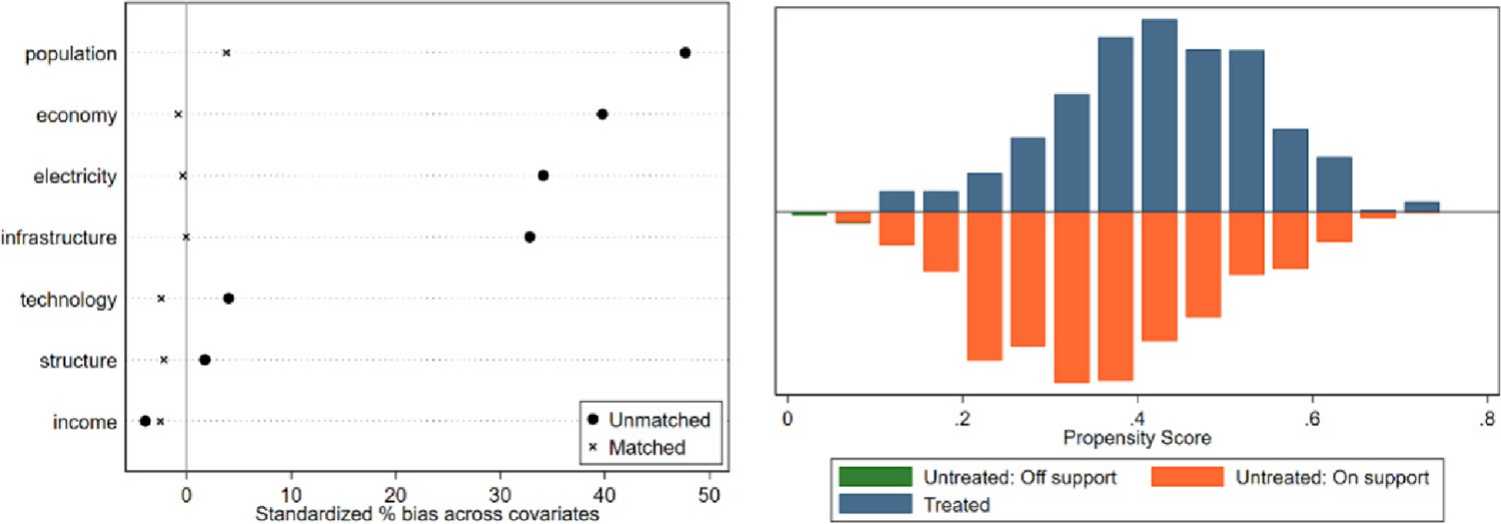
Kernel matching equilibrium test and PS plot.

In this paper, a counterfactual research sample was constructed using the PSM method, and its findings were used as evidence for robust type testing. The samples from non-overlapping regions are dropped out to satisfy the common support hypothesis, and the balance test of matching variables is conducted. The test results are shown in Fig 5. The matching balance test results show that the standard deviations of the main variables of the treatment and control groups after matching are less than 10%., indicating that the matching the matched estimation results are valid and reliable. In addition, this paper adds two additional estimation methods, radius matching, and kernel matching, to the nearest neighbor matching. The balance tests of these two matching methods and the results of PS plots are shown in Figs 6 and 7. The ETS estimation results obtained by these three matching methods are shown in Table 8. From Table 8, the effect of ETS on SO_2_ is significantly negative at the 1% level, indicating that there is a significant suppression effect of ETS on SO_2_. This result is consistent with the results of the benchmark regression, which further enhances the robustness of the empirical results.

**Table 8:** PSM-DID estimation results.

	(1)	(2)	(3)
VARIABLES	SO_2_	SO_2_	SO_2_
**EST**	-0.2584***	-0.2664***	-0.2582***
	(0.0420)	(0.0421)	(0.0431)
**Economy**	1.0242***	0.9981***	0.6553***
	(0.2293)	(0.2294)	(0.2336)
**Structure**	-0.0045*	-0.0049**	-0.0103***
	(0.0024)	(0.0024)	(0.0023)
**Population**	-0.0793	-0.0796	-0.0796
	(0.0538)	(0.0539)	(0.0555)
**Electricity**	-0.0937***	-0.0923***	-0.0941***
	(0.0132)	(0.0133)	(0.0135)
**Income**	-0.8620***	-0.8724***	-0.5900**
	(0.2251)	(0.2253)	(0.2303)
**Infrastructure**	0.2214***	0.2245***	0.2186***
	(0.0324)	(0.0326)	(0.0332)
**Technology**	-0.0045**	-0.0050**	-0.0050**
	(0.0021)	(0.0021)	(0.0021)
**Constant**	12.3469***	12.6112***	12.1550***
	(1.3008)	(1.3059)	(1.3386)
**Year**	Yes	Yes	Yes
**Province**	Yes	Yes	Yes
**Observations**	4,210	4,219	4,267
**R-squared**	0.5418	0.5382	0.5209

Standard errors in parentheses

*** p<0.01

** p<0.05

* p<0.1

#### 5.5.2 Placebo test

In the placebo test, counterfactual tests were conducted in this paper from both implementation area and implementation time perspectives, and the results of the tests are shown in Table 9. Table 9-(1) shows the results of the counterfactual test for the implementation time, and we can see that implementing ETS at non-actual pilot times significantly promoted SO_2_ emissions, which is opposed to the baseline regression results. Table 9-(2) shows the results of the counterfactual test for the implementation area, and it is found that ETS in the non-pilot implementation region has a significant contribution effect on SO_2_. This result is opposed to the baseline regression results. Therefore, the placebo test conducted for the implementation region passed. The robustness of the empirical results is further enhanced.

**Table 9:** Placebo test results.

	(1)	(2)
VARIABLES	SO_2_	SO_2_
**ETS(-3)**	0.3148***	
	(0.0696)	
**ETS(-2)**	0.2609***	
	(0.0696)	
**ETS(-1)**	0.2176***	
	(0.0695)	
**ETS**		0.2616***
		(0.0433)
**Economy**	0.6546***	0.6554***
	(0.2340)	(0.2340)
**Structure**	-0.0092***	-0.0093***
	(0.0023)	(0.0023)
**Population**	-0.0763	-0.0755
	(0.0556)	(0.0556)
**Electricity**	-0.0943***	-0.0942***
	(0.0135)	(0.0135)
**Income**	-0.6108***	-0.6094***
	(0.2308)	(0.2308)
**Infrastructure**	0.2182***	0.2176***
	(0.0333)	(0.0333)
**Technology**	-0.0045**	-0.0045**
	(0.0021)	(0.0021)
**Constant**	12.1769***	12.2785***
	(1.3402)	(1.3407)
**Year**	Yes	Yes
**Province**	Yes	Yes
**Observations**	4,275	4,275
**R-squared**	0.5185	0.5182

Standard errors in parentheses

*** p<0.01

** p<0.05

* p<0.1

#### 5.5.3 Removal of policy interference

**Fig 8:**
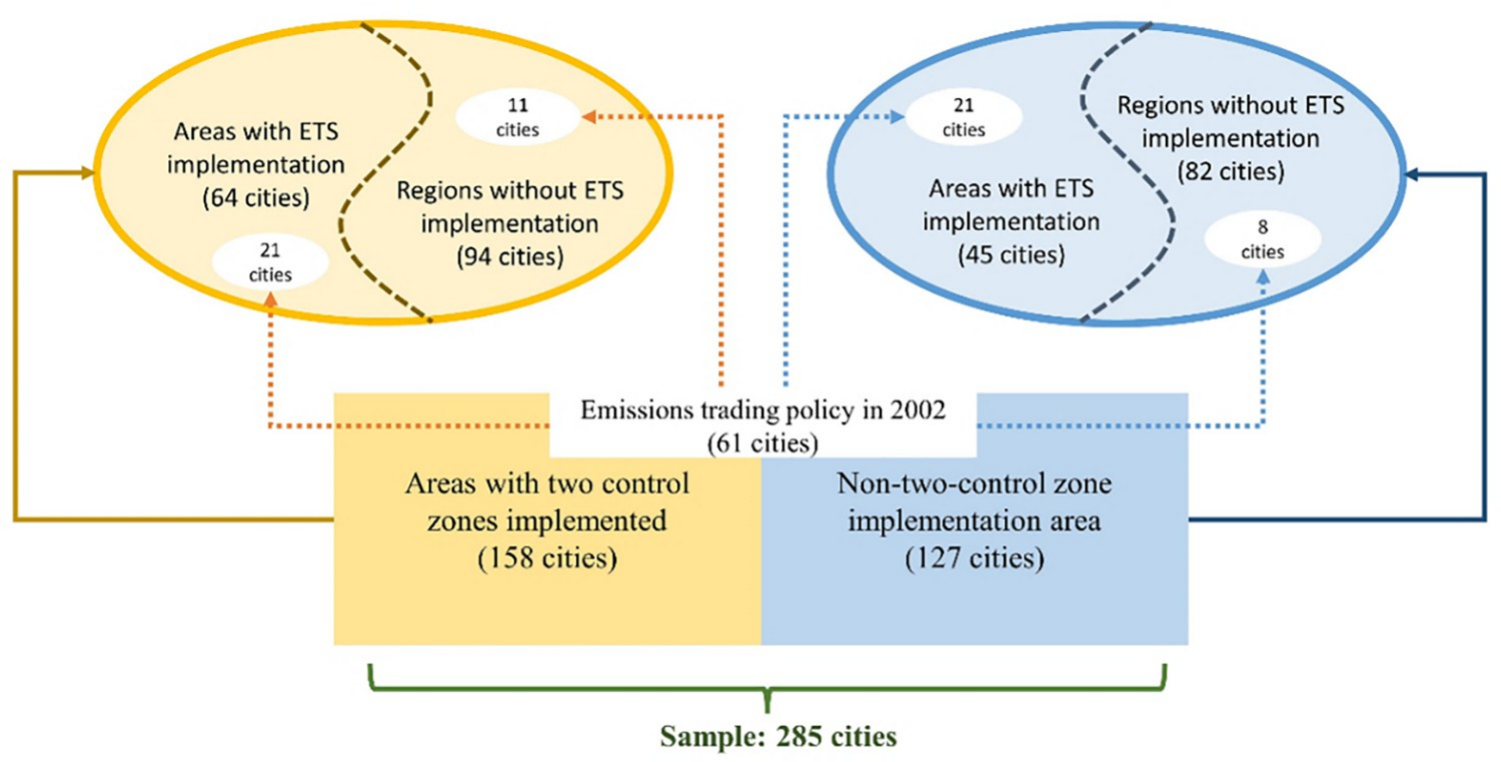
Grouping idea of removing policy interference.

In the robustness test excluding other policy interferences, this paper excludes representative policies affecting SO_2_ emissions and thus observes the approximate effect of ETS alone. The two control zones in 2000 and the pilot emissions trading scheme in 2002 were selected as the "interfering policies." In 2000 the government implemented two control zones to control acid rain formation and SO_2_ pollution. The total area of China’s two control areas is about 1.09 million square kilometers. The acid rain control area is approximately 800,000 square kilometers, and the sulfur dioxide pollution control area is about 290,000 square kilometers. And before 2007, as early as 2002, China had implemented the emissions trading policy in four provinces (Shandong Province, Shanxi Province, Jiangsu Province, and Henan Province) and three cities (Shanghai, Tianjin, and Liuzhou City). Due to the large area occupied by the two control areas, to prevent estimation bias, this paper divides the model into two parts according to the implementation of the two control areas. The specific grouping idea is shown in Fig 8. The obtained test results are shown in Table 10. Table 10-(1) shows the policy effect of ETS in the implementation area of the two control areas, and Table 10-(2) shows the policy effect of ETS in the implementation area of the non-two control areas. Table 10-(3) shows the effect of ETS for the single exclusion of the 2002 Emissions Trading Policy Pilot Area. Table 10-(4) shows the policy effect of ETS after excluding the 2002 emissions trading policy pilot based on the two control areas implementation areas. And Table 10-(5) shows the policy effect of ETS after excluding the 2002 emissions trading policy pilot based on the non-two control areas implementation areas. From Table 10, we can find that ETS has a significant suppression effect on SO_2_ emissions regardless of the way of grouping. This result is consistent with the results of the benchmark regression, which further enhances the robustness of the empirical results. Meanwhile, from the test results in Table 9, it is found that the suppression effect of ETS on SO_2_ is more significant in the two control areas than in the non-two control areas after excluding the 2002 emissions trading policy. Since the two control areas have been aware of SO_2_ emission reduction since 1998, they have relatively rich experience and infrastructure for SO_2_ management. Therefore, after implementing ETS, the two control areas can use the existing resources to achieve SO_2_ emission reduction more effectively.

**Table 10:** Estimated results of the removal of interference policy (1).

	(1)	(2)	(3)	(4)	(5)
VARIABLES	SO_2_	SO_2_	SO_2_	SO_2_	SO_2_
**ETS**	-0.2436***	-0.2837***	-0.2630***	-0.2483***	-0.2390**
	(0.0500)	(0.0744)	(0.0530)	(0.0582)	(0.0979)
**Economy**	1.0154***	-0.2805	0.7443***	1.2073***	-0.2177
	(0.2561)	(0.4673)	(0.2477)	(0.2685)	(0.4970)
**Structure**	-0.0083***	-0.0108***	-0.0043	-0.0055	-0.0045
	(0.0031)	(0.0035)	(0.0027)	(0.0036)	(0.0040)
**Population**	-0.1900***	0.1029	-0.0411	-0.1923***	0.3596**
	(0.0599)	(0.1069)	(0.0677)	(0.0677)	(0.1482)
**Electricity**	-0.1183***	-0.0591***	-0.0890***	-0.1232***	-0.0430*
	(0.0181)	(0.0209)	(0.0154)	(0.0204)	(0.0237)
**Income**	-0.6706***	0.1354	-0.5977**	-0.5291**	0.0870
	(0.2410)	(0.4824)	(0.2431)	(0.2506)	(0.5138)
**Infrastructure**	0.1012**	0.3034***	0.1815***	0.0386	0.2736***
	(0.0416)	(0.0532)	(0.0373)	(0.0456)	(0.0611)
**Technology**	-0.0050	-0.0025	-0.0050**	-0.0028	-0.0039
	(0.0033)	(0.0029)	(0.0023)	(0.0036)	(0.0031)
**Constant**	12.0643***	8.8026***	11.3516***	9.6349***	7.1670**
	(1.5771)	(2.5351)	(1.4841)	(1.7483)	(2.8615)
**Year**	Yes	Yes	Yes	Yes	Yes
**Province**	Yes	Yes	Yes	Yes	Yes
**Observations**	2370	1905	3360	1,890	1,470
**R-squared**	0.6320	0.4087	0.4774	0.6154	0.3492

Standard errors in parentheses

*** p<0.01

** p<0.05

* p<0.1

After implementing ETS in 2007, China implemented the carbon emission trading policy (CES) in 2013, and ZhiQing Dong et al. (2020) showed that this policy also has a significant effect on SO_2_ emissions [76]. Therefore, this paper uses the same idea to test the effect of ETS on SO_2_ after excluding CES, and the test results are shown in Table 11. Table 11-(1) shows the effect of ETS on SO_2_ after excluding CES pilot areas; Table 11-(2) shows the effect of ETS on SO_2_ after excluding the 2002 pilot emissions trading policy based on Table 11-(1). Table 11-(3) shows the effect of ETS on SO_2_ in the two control areas based on Table 11-(2). Table 11-(4) shows the effect of ETS on SO_2_ in the non-two control areas based on Table 11-(2). From the results in Table 11, it is found that ETS still has a significant suppression effect on SO_2_ emissions after continuously removing the disturbance policy, and this result is consistent with the results of the benchmark regression, which further enhances the robustness of the empirical results.

**Table 11:** Estimated results of the removal of interference policy (2).

	(1)	(2)	(3)	(4)
VARIABLES	SO_2_	SO_2_	SO_2_	SO_2_
**ETS**	-0.2170***	-0.1898***	-0.1970***	-0.1861*
	(0.0467)	(0.0591)	(0.0645)	(0.0984)
**Economy**	0.4182	0.4542	0.5089	0.3052
	(0.2745)	(0.2937)	(0.3164)	(0.5717)
**Structure**	-0.0103***	-0.0054*	-0.0028	-0.0055
	(0.0025)	(0.0029)	(0.0042)	(0.0040)
**Population**	-0.1213*	-0.0934	-0.3131***	0.3594**
	(0.0723)	(0.1032)	(0.0986)	(0.1642)
**Electricity**	-0.0672***	-0.0577***	-0.0722***	-0.0334
	(0.0146)	(0.0169)	(0.0232)	(0.0239)
**Income**	-0.5344**	-0.4360	-0.1212	-0.5344
	(0.2723)	(0.2898)	(0.2940)	(0.5929)
**Infrastructure**	0.2307***	0.1995***	0.0576	0.2924***
	(0.0360)	(0.0410)	(0.0512)	(0.0625)
**Technology**	-0.0059***	-0.0068***	-0.0050	-0.0038
	(0.0022)	(0.0024)	(0.0039)	(0.0031)
**Constant**	13.2757***	11.8351***	10.6591***	10.2231***
	(1.5002)	(1.6920)	(1.9340)	(3.1742)
**Year**	Yes	Yes	Yes	Yes
**Province**	Yes	Yes	Yes	Yes
**Observations**	3705	2,835	1,500	1,335
**R-squared**	0.5680	0.4527	0.6185	0.3491

Standard errors in parentheses

*** p<0.01

** p<0.05

* p<0.1

### 5.6 Heterogeneity analysis

#### 5.6.1 Heterogeneous impact of east, central and west

The above analysis verified the policy effect of ETS on SO_2_ emission reduction in China from the national level, but China is a developing country with uneven development, and there is heterogeneity in policy implementation at the regional level. Therefore, this paper analyzed cities in the east, central and west separately according to the classification criteria of the National Bureau of Statistics, and the test results are shown in Table 12. From Table 12, it can be seen that there is regional heterogeneity in the suppression effect of ETS on SO_2_, from strong to weak in the order of eastern, central, and western.

**Table 12:** Heterogeneous impact of east, central and west.

	Eastern	Center	Western
VARIABLES	SO_2_	SO_2_	SO_2_
**ETS**	-0.2583***	-0.1857***	-0.1671
	(0.0750)	(0.0610)	(0.1267)
**Economy**	0.8442***	-0.8857	-0.6190
	(0.3080)	(1.0219)	(0.5170)
**Structure**	-0.0248***	-0.0079**	0.0094*
	(0.0044)	(0.0035)	(0.0049)
**Population**	-0.1399	-0.0377	0.8911*
	(0.0885)	(0.0661)	(0.4712)
**Electricity**	-0.1953***	-0.0445**	-0.0525**
	(0.0263)	(0.0191)	(0.0267)
**Income**	-0.7355**	0.4094	0.4364
	(0.3005)	(1.0207)	(0.4925)
**Infrastructure**	0.0645	0.2061***	0.2693***
	(0.0700)	(0.0426)	(0.0762)
**Technology**	-0.0086*	-0.0040	-0.0039
	(0.0048)	(0.0036)	(0.0032)
**Constant**	13.9605***	11.4141***	2.8476
	(2.2375)	(3.9434)	(4.1582)
**Year**	Yes	Yes	Yes
**Province**	Yes	Yes	Yes
**Observations**	1,725	1,635	915
**R-squared**	0.5390	0.6098	0.4054

Standard errors in parentheses

*** p<0.01

** p<0.05

* p<0.1

The eastern, central, and western regions have different industrial structures due to their geographical locations and energy endowments. The east part can better introduce advanced talents and resources in economic development with its inherent location advantage and establish the concept of green and sustainable development by contacting the new green industries earlier than the central and western regions. In ETS promotion, the eastern part can effectively use this resource advantage to achieve SO_2_ emission reduction. The central area will also enjoy the advanced resources of the eastern region due to its location close to the east region. However, compared with the east area, the central area has a relatively large proportion of industry and a vital resource dependency, and the amount of SO_2_ emissions are relatively high. In the process of ETS implementation, it can make better use of the existing advanced resources and actively make technical and technological innovations to effectively suppress regional SO_2_ emissions. The western region is located inland. Its inherent geographical disadvantages make it rank at the back of the country in terms of labor resources, knowledge content, openness level, foreign trade dependence, fixed capital stock, etc. These objective conditions limit the promotion of the concept of green development in the region. Still, the resources are vibrant, such as Xinjiang, Gansu, Qinghai is a wealthy region of China’s oil resources, and the northwest region has 38% of China’s power coal, etc. The advantageous prerequisite conditions of factor endowment make these regions have low factor utilization rate and severe environmental pollution due to the mismatch of technology level and industrial layout in production and processing. In recent years, the final demand for construction and heavy industry in less developed provinces in central and western China has caused an increase in SO_2_ emissions [77]. In addition, under the strategy of industrial location transfer, the Western region has undertaken more traditional high pollution type industries from home and abroad. Therefore, ETS did not significantly suppress SO_2_ emissions in the western region.

#### 5.6.2 Heterogeneity analysis of different types of resource-based cities

Resource-based cities are cities with the mining and processing of natural resources such as minerals and forests in the region as the leading industry. It is an essential strategic guarantee base for energy resources in China. The National Plan for Sustainable Development of Resource-based Cities (2013–2020) identifies 126 resource-based cities and classifies resource-based cities into four types: growing, mature, declining, and regenerating, according to their resource security capacity and sustainable economic and social development capacity. According to the principle of factor endowment, resource-based cities have a certain impact on the environment with their unique, innate advantages. Therefore, we analyze the heterogeneity of the effect of ETS on SO_2_ from the perspective of resource-based cities, and the test results are shown in Table 13. Table 13-(1) to Table 13-(6) show the DID test results for all resource-based cities, growing cities, mature cities, declining cities, regenerating cities, and non-resource-based cities. From Table 13, we can see that ETS has a significant suppression effect on SO_2_ in both resource-based and non-resource-based cities, and the impact in non-resource-based is more potent than that of resource-based cities. In addition, among resource-based cities, ETS implementation has a significant suppression effect only on mature cities.

**Table 13:** Heterogeneity analysis of different types of resource-based cities.

	(1)	(2)	(3)	(4)	(5)	(6)
VARIABLES	SO_2_	SO_2_	SO_2_	SO_2_	SO_2_	SO_2_
**ETS**	-0.2293***	-0.2030	-0.1369	0.0388	-0.6194***	-0.2981***
	(0.0631)	(0.2060)	(0.0843)	(0.1698)	(0.1823)	(0.0588)
**Economy**	1.4514***	4.9364***	0.5687	-1.7049	-5.4767*	0.4608
	(0.4328)	(1.2774)	(0.5067)	(1.1380)	(3.0713)	(0.2968)
**Structure**	-0.0019	0.0036	-0.0020	-0.0164**	-0.0347***	-0.0155***
	(0.0032)	(0.0094)	(0.0042)	(0.0079)	(0.0113)	(0.0033)
**Population**	-0.0431	2.2567*	-0.0193	-0.1023	-0.4503	-0.0882
	(0.0812)	(1.3496)	(0.0979)	(0.1584)	(0.2782)	(0.0751)
**Electricity**	-0.0557***	-0.0431	-0.0855***	0.2336***	0.0110	-0.1276***
	(0.0185)	(0.0519)	(0.0225)	(0.0881)	(0.0743)	(0.0193)
**Income**	-1.4151***	-5.0673***	-0.6204	0.3684	5.3101*	-0.3262
	(0.4321)	(1.3742)	(0.5052)	(1.0952)	(2.9628)	(0.2846)
**Infrastructure**	0.1869***	0.3023**	0.2596***	-0.0808	0.4964**	0.2403***
	(0.0467)	(0.1333)	(0.0678)	(0.0901)	(0.1964)	(0.0480)
**Technology**	-0.0084***	-0.0094	-0.0107**	-0.0023	-0.0260***	-0.0014
	(0.0032)	(0.0124)	(0.0051)	(0.0063)	(0.0080)	(0.0029)
**Constant**	15.5771***	18.4862*	12.6996***	17.5114***	-4.0715	10.7254***
	(2.1187)	(10.3537)	(2.7205)	(5.2309)	(10.2503)	(1.9688)
**Year**	Yes	Yes	Yes	Yes	Yes	Yes
**Province**	Yes	Yes	Yes	Yes	Yes	Yes
**Observations**	1,725	225	930	345	225	2550
**R-squared**	0.5449	0.4867	0.5869	0.6156	0.5979	0.5090

Standard errors in parentheses

*** p<0.01

** p<0.05

* p<0.1

The explanations for this phenomenon in this paper are as follows. First, the cause of the differential effect of ETS between resource-based cities and non-resource-based cities. Resource-based cities are resource-rich and based on factor endowment theory. Their production is more resource-dependent than that of non-resource-based cities. In addition, according to Hotelling’s law [78], the cost of extracting some resources will increase in the future because of their non-renewable nature. The introduction of ETS conveys the value of clean production to production units, and energy suppliers will extract energy early and sell it at a low price, considering that clean energy will occupy a larger market in the future. Low-priced energy will attract energy-dependent industries and thus hinder the process of SO_2_ reduction by ETS. Therefore, compared with resource-based cities, non-resource-based cities have more potent emission reduction effects. Second, ETS only has a significant suppression effect on SO_2_ in regenerative cities among resource-based cities. Regenerative cities are free from resource dependence. Their economy and society have started to enter a virtuous development track, and they are the pioneer area for resource-based cities to transform their economic development model. Therefore, in ETS implementation, regenerative cities can take advantage of their advantages to speed up the move away from energy dependence and find clean production methods, thus significantly curbing SO_2_ emissions. Growing cities are in the rising stage of resource development, while mature cities are in the stable stage of resource development. They have the characteristics of a high potential for resource security and substantial economic and social development. These two types of cities are at the stage of solid resource dependence, high resource consumption, and abundant resource supply. Therefore, ETS has a suppressive effect on SO_2_ emission reduction in these two types of cities, but it is not apparent. Since cities in the growth stage are smaller in industrial scale and more accessible to transform than cities in the mature stage, the suppression effect in the growth stage is better than that in the mature stage when the suppression effect is not apparent. Declining cities have entered the declining resource development phase, with stronger resource dependence but insufficient resource supply. Unlike mature cities, declining cities missed the critical period of transformation, and ETS did not suppress SO_2_ reduction in declining cities because of the difficulties in SO_2_ reduction due to infrastructure and industrial accumulation.

#### 5.6.3 Heterogeneity analysis of old industrial base cities

Old industrial bases are industrial areas with relatively complete and concentrated categories formed by relying on state investment and construction during the planned economy. The National Plan for the Adjustment and Transformation of Old Industrial Bases (2013–2022) identifies 120 old industrial base cities. Some of the old industrial bases are important national energy bases and usually undertake the supply of major technical equipment or products related to the people’s livelihood. Therefore, this paper analyzes the heterogeneity of the effect of ETS on SO_2_ from the perspective of old industrial bases, and the test results are shown in Table 14. Table 14-(1) shows the test results of old industrial bases, and Table 14-(2) shows the test results of non-old industrial bases. From the test results, we can find that ETS has a significant suppression effect on SO_2_ regardless of whether it is an old industrial base. In comparison, this effect is stronger for non-old industrial bases than for old industrial bases.

**Table 14:** Heterogeneity analysis of old industrial base cities.

	(1)	(2)
VARIABLES	SO_2_	SO_2_
**ETS**	-0.2169***	-0.2678***
	(0.0575)	(0.0616)
**Economy**	-0.3044	0.8270***
	(0.4336)	(0.2921)
**Structure**	0.0061*	-0.0170***
	(0.0034)	(0.0032)
**Population**	-0.0896	-0.0782
	(0.0798)	(0.0751)
**Electricity**	-0.0255	-0.0877***
	(0.0228)	(0.0179)
**Income**	0.1318	-0.6038**
	(0.4307)	(0.2865)
**Infrastructure**	0.1940***	0.2294***
	(0.0437)	(0.0482)
**Technology**	-0.0044	-0.0016
	(0.0032)	(0.0029)
**Constant**	11.0688***	11.1378***
	(2.0151)	(1.9344)
**Year**	Yes	Yes
**Province**	Yes	Yes
**Observations**	1,800	2,475
**R-squared**	0.6389	0.4464

Standard errors in parentheses

*** p<0.01

** p<0.05

* p<0.1

This result is explained as follows. The old industrial bases within the planning area are characterized by a robust industrial base, large industrial scale, abundant natural resources, and high technological potential, and their development conditions are good. However, their development methods are still relatively crude. The long-accumulated industrial structure and production mode make the old industrial bases more energy and technology-dependent, characterized by high energy consumption and high pollution. As an essential pillar of urban economic development, the region used to have relatively few demands on environmental quality. The implementation of ETS has made it necessary for cities in old industrial bases to control emissions while ensuring their development, which has brought pressure on emissions from old industrial bases and made them less motivated to participate in emissions trading. According to data analysis, the SO_2_ emission intensity of old industrial bases is 1.5 times the national average, respectively. Old industrial bases are the key transformation areas to achieve SO_2_ reduction at the national level. Therefore, the state gives subsidies and other preferential policies to the old industrial bases to mobilize them to participate in ETS and help them change their production methods to realize industrial upgrading, thus making SO_2_ emissions reduction. In contrast, non-old industrial bases are mostly economic development areas with more environmental quality claims and higher motivation to participate in emissions trading. In addition, non-old industrial bases have relatively lower industrial energy dependence and more vital awareness of cleaner production in industrial development. Therefore, ETS can significantly suppress SO_2_ emissions from old and non-old industrial bases. The suppression effect of non-old industrial bases is more potent than that of old industrial bases.

### 5.7 Discussion

The empirical part of this paper, 5.1 to 5.4, is the confirmation part of the four hypotheses in this paper, and from 5.5 is the robustness test, which further confirms the robustness of the research results through the PSM-DID, placebo test, and exclusion of interference policy test. This paper makes the following statement for each hypothesis in this paper.

Hypothesis 1: Emissions trading policy can effectively reduce SO_2_ emissions. This hypothesis is confirmed in the baseline regression of 5.2, while the parallel trend test of 5.3 further strengthens the reliability of this research conclusion. Since ETS is an environmental regulation tool, internal sources transfer their emissions to each other through the monetary exchange, thus reducing emissions. This linking emissions to cost-effectiveness motivate enterprises to participate and thus achieve SO_2_ emission reduction.

Hypothesis 2: Emissions trading policy promotes SO_2_ reduction by reducing government intervention. This hypothesis is confirmed by the estimation of the mediating effect of government intervention in 5.4.1. ETS not only releases some of the government’s environmental regulation power but also relieves some of the pressure on government financial subsidies by establishing a trading market and circulating pollution shares as commodities in the market. So enterprises can realize their environmental awareness through trading emission shares. It not only releases some of the government’s environmental regulatory power but also relieves some of the pressure on government financial subsidies.

Hypothesis 3: Emissions trading policy can achieve SO_2_ emission reduction by promoting technological innovation, confirmed by the estimated results of 5.4.2 on technological innovation. ETS is a policy traded so that environmentally conscious units can gain more revenue and thus invest in technological R&D. The government subsidizes units that change their processes and production in the direction of environmental protection, which stimulates innovative research and development in the field of environmental protection.

Hypothesis 4: Emissions trading policy promotes SO_2_ emission reduction by improving energy efficiency, and this hypothesis is confirmed by the estimated intermediary effect of energy use efficiency in 5.4.3. The environmental pressure brought by ETS can make enterprises pay more attention to energy use efficiency, which greatly avoids energy waste and can better promote the rational and effective distribution of energy among production units. Thus SO_2_ reduction is achieved.

In addition, due to regional differences, heterogeneity analysis is conducted in this paper in 5.6. In this paper, two types of heterogeneity analysis are conducted for resource-based cities and non-resource-based cities, and industrial bases and non-industrial bases. From the heterogeneity analysis, the resource endowment and the advantage of the regional industrial base will make the industry the pillar industry of the region, which will also pressure the region’s environment. Therefore, it will generate ETS in the region’s resource endowment or good industrial base to promote relatively more complexity.

Compared with the previous research results, this paper has three breakthroughs while confirming some of their research results. First, the research sample is urban. Second, the research method is a detailed robustness test, which is neglected in the previous research results. Third, the research perspective is that existing scholars analyze the transmission path of ETS to SO_2_ from two perspectives: energy and government. Furthermore, heterogeneity analysis is conducted from two perspectives: resource-based cities and industrial cities, which enriches the research in the field of ETS to some extent, enriching the research in the field of ETS to a certain extent.

Compared with the previous research results, this paper has three breakthroughs while confirming some of their research results. First, the research sample is urban. Second, the research method is a detailed robustness test, which is neglected in the previous research results. Third, the research perspective is that existing scholars analyze the transmission path of ETS to SO_2_ from two perspectives: energy and government. Furthermore, heterogeneity analysis is conducted from two perspectives: resource-based cities and industrial cities, which enriches the research in the field of ETS to some extent, enriching the research in the field of ETS to a certain extent.

## 6 Conclusions

This paper examines the effect of ETS on SO_2_ emissions in China and the mechanism of influence based on sample data from 285 cities (except Tibet) from 2004 to 2018. The research conclusions obtained from this paper are as follows. First, ETS has a significant suppression effect on SO_2_ in China, and the results still hold after the parallel trend test as well as the placebo test. Second, the mechanism test shows that ETS can effectively suppress SO_2_ by reducing GOV, stimulating GTIA, and improving RUE, while GTIB has a masking effect in it. Third, in terms of the East, Central, and West regions, ETS has a significant suppression effect on SO_2_ emissions in the Eastern and Central regions. The Eastern part is better than the Central region. Fourth, in terms of factor endowment, ETS has a significant suppression effect on SO_2_ in both resource-based and non-resource-based cities, with non-resource-based cities outperforming resource-based cities. While within resource-based cities, this effect exists only in regenerative cities. Fifth, in terms of industrial base zoning, ETS has a significant suppression effect on SO_2_ in both old industrial base cities and non-old industrial base cities. The suppression effect in non-old industrial base cities is better than that in old industrial base cities.

Based on the above findings, this paper puts forward targeted policy recommendations on the specific challenges faced by China’s ETS to curb SO_2_ emissions at this stage.

First, ETS is an essential environmental regulation tool for managing SO_2_ emissions. The government should improve the system design of pricing and quotas to improve the efficiency of market transactions. At the same time, it should enhance the price regulation mechanism and rationalize the pricing of trading shares to provide a good market environment and a trading platform for the implementation of ETS. In addition, the government should gradually expand the scope of markets and subjects of the emissions trading system to include more pollutants in the matter of trading.

Secondly, in terms of government intervention, the government should improve the market mechanism and strengthen the regulation of the market. On this basis, it should strengthen information disclosure, promote healthy competition, and reduce unnecessary market intervention. ETS can give full play to the role of the market mechanism in environmental governance.

Thirdly, in terms of the green technology innovation path, the government should strengthen the protection of intellectual property rights, establish a green technology standard system, the entire life cycle management of products, and enhance risk identification and control. Create a good innovation environment. At the same time, it should play the role of government incentives to motivate enterprises to actively participate, increase their R&D investment, and develop new low-pollution and clean technologies to improve green innovation. Increase subsidies for units that make outstanding contributions in the field of GTI.

Fourthly, in terms of resource utilization efficiency, strengthen the regulation of the energy supply industry and energy prices, encourage energy supply units to adjust their energy supply structure, and prevent vicious exploitation by energy suppliers. And under the premise of controlled environmental risks, we will implement targeted utilization for energy-consuming enterprises and encourage production units to reduce energy dependence. At the same time, fully develop cooperation with universities and research institutes, vigorously introduce relevant technologies and talents in environmental protection, and encourage and guide social funds to participate in the implementation of ETS.

Fifthly, in response to the phenomenon of uneven regional development, the government should pay attention to the differences in SO_2_ emission reduction effects in different regions during ETS implementation, fully consider each region’s development characteristics, and promote policy implementation in a targeted manner. In terms of the East, Central and West, the government should actively guide the flow of capital into the western region and encourage the area of the west to undertake new industrial models. Develop concepts to accelerate the adaptation of technology levels to regional industrial structures and thus make more effective use of its rich natural factor endowment advantages. For resource-based cities, the government should encourage regenerative cities to eliminate energy dependence and promote new industries. Help mature and growing cities to realize economic transformation actively and guide declining cities to introduce low-pollution and high-value-added industries. For old industrial bases, strengthen ETS implementation for old industrial bases with high pollution. Encourage them to use their own scientific and technological potential to accelerate the transformation of achievements and realize the change in production methods.
